# Cellular Reference Materials for DNA Damage Using Electrochemical Oxidation

**DOI:** 10.1155/2020/2928104

**Published:** 2020-01-30

**Authors:** Donald H. Atha, Omobola Cole, Breece Clancy, Alessandro Tona, Vytas Reipa

**Affiliations:** ^1^Materials Measurement Laboratory, Biosystems and Biomaterials Division, National Institute of Standards and Technology, Gaithersburg, Maryland 20899, USA; ^2^Department of Biochemistry & Molecular and Cellular Biology, Georgetown University Medical Center, Washington, DC 20007, USA; ^3^Montgomery College, Germantown, Maryland 20876, USA

## Abstract

Reference materials are needed to quantify the level of DNA damage in cells, to assess sources of measurement variability and to compare results from different laboratories. The comet assay (single cell gel electrophoresis) is a widely used method to determine DNA damage in the form of strand breaks. Here we examine the use of electrochemical oxidation to produce DNA damage in cultured mammalian cells and quantify its percentage using the comet assay. Chinese hamster ovary (CHO) cells were grown on an indium tin oxide electrode surface and exposed 12 h to electrochemical potentials ranging from 0.5 V to 1.5 V (vs Ag/AgCl). The resulting cells were harvested and analyzed by comet and a cell viability assay. We observed a linear increase in the percentage (DNA in tail) of strand breaks along with a loss of cell viability with increasing oxidation potential value. The results indicate that electrochemically induced DNA damage can be produced in mammalian cells under well-controlled conditions and could be considered in making a cellular reference material for the comet assay.

## 1. Introduction

Exposure of mammalian cells to environmental factors, such as toxic pollutants, ionizing and UV radiation can have genotoxic consequences. Modification of genomic DNA by the reactive oxygen species (ROS) catalyzed by these factors is implicated in the development of mutagenesis, carcinogenesis, and pathogenesis of numerous diseases including AIDS, Huntington's, Parkinson's, and Alzheimer's. Cells undergo oxidative stress when they are exposed to oxidative outbreaks that exceed their capability to compensate with internal antioxidants, such as glutathione, catalase or superoxide dismutase. This can lead to many severe outcomes, which include peroxidation of cellular proteins and lipids, disruption of metabolic functions such as mitochondrial activity and DNA damage [1]. Oxidative DNA damage leads to many types of structural perturbations. These include DNA base lesions such as 8-hydroxyguanine as well as strand breaks. Measurement of DNA damage allows the detection of the genotoxicity at an early stage [2, 3]. Single cell gel electrophoresis (SCGE), also known as comet assay, is a sensitive method that can be used to detect clinically relevant levels of DNA damage [4–6], and is widely used for genotoxicity testing [7]. Cells undergoing the comet assay are imbedded in an agarose gel on microscope slides, followed by lysis, denaturation and electrophoresis, which allows broken DNA strands to migrate outside the nucleus. Following staining with a DNA binding dye, the resulting comet-like patterns are analyzed with a microscope and quantified using densitometric and image analysis procedures [3, 8].

The comet assay, however, has been known to suffer from significant experimental variability from lab to lab, traceable to changes in certain steps of the procedure [3, 9]. In a previous study, we examined the role of the microscope imaging and analysis on the assay output variation [10]. Attempts have been made to reduce comet assay variation by adopting a completely standardized protocol but identification of one that is universally accepted using equivalent reagents and instrumentation remains to be established [11, 12]. Additional efforts include the use of cells that were intentionally subjected to a genotoxic environment and could be utilized as calibrants during the comet assay procedure [13, 14].

In a preceding investigation we utilized an electrochemical potential gradient as an *in vitro* platform for simulating the cellular oxidative stress [15]. In that study, we assayed viability in the cultured mammalian cells in a redox potential gradient and found that the electrochemical oxidation mimics oxidative stress and could be used to test the effect of antioxidants. In a separate study, a controlled potential preparative electro-oxidation of soluble calf thymus DNA produced DNA lesions,that were quantified by gas chromatographic mass spectrometry (GC/MS/MS) [16]. In the current report, we examine the use of electrochemical oxidation to produce a controlled amount of DNA strand breaks in cultured mammalian cells. We envision that after such treatment cells could be utilized as cellular genomic DNA reference materials that may facilitate calibration of genotox assays as well as other potential applications.

## 2. Materials and Methods

Stock cultures of Chinese hamster ovary CHO K1 cells (ATCC, Manassas, VA, USA) were grown at 37°C, 5% CO_2_ and 95% relative humidity in Iscove's modified Dulbecco's modified medium, IMDM (Gibco, Carlsbad, CA), 10% (v/v) fetal bovine serum, FBS (Gibco), 1% (v/v), penicillin-streptomycin (100 units/mL, and 100 *µ*g/mL).

### 2.1. Electrochemical Oxidation of Live Cells

A uniform oxidative treatment was applied by growing the CHO cells on a working electrode surface polarized at a fixed positive potential. Indium tin oxide (InSnO_2_) films on glass (Delta Technologies, Loveland, CO) were used as transparent electrodes (5 cm × 8 cm), placed in 140 mm diameter plastic Petri dishes with 0.5 mm Pt wire as a counter electrode and a Ag/AgCl reference electrode (Microelectrodes, Inc.). Working electrodes were cleaned by sonicating 15 min in hot water and ethanol mixture, followed by air drying prior to mounting them in Petri dishes. Contacts to the conducting film surface were provided by wire attached via InGa eutectic and insulated with a waterproof silicone. Electrode surfaces were treated for 1 h at room temperature with 25 *µ*g/mL bovine fibronectin (Sigma-Aldrich, St. Louis, MO) in Dulbecco's phosphate buffered saline, DPBS (Gibco, Carlsbad, CA), to facilitate adhesion. At first cells (seeded ≈ 10^6^cells in 40 mL growth medium, 8 × 10^3^ cells/cm^2^) were grown at 37°C, 5% CO_2_ and 95% relative humidity in complete growth medium (above) on the InSnO_2_ electrodes at open circuit potential (≈−0.1 V) until they reached confluence (≈3 days), then electrode potentials (E) at 0.5 V, 1.0 V and 1.5 V (vs Ag/AgCl) were applied for 12 h using EG&G Model 263 potentiostat along with an open circuit control for 12 h. Following oxidative treatment, electrodes were gently rinsed with DPBS and placed in a clean Petri dish. Trypsin EDTA (2 mL of 2.5 mg/mL) was added for a few minutes at 37°C until complete cell detachment from the electrode surfaces. The trypsin treatment was stopped with the addition of 10 mL Dulbecco's modified Eagle's medium, 10% (v/v) FBS. The cells from the entire electrode surface, at each treatment level, were collected separately, concentrated by centrifugation at 250 × g for 5 min at 4°C and resuspended in DPBS.

### 2.2. Live/Dead Assay

Cell growth conditions were maintained during the electrochemical treatment (37°C, 5% CO_2_ and 90% relative humidity). Immediately after terminating the potential application, the growth media was removed, electrode plate slides were rinsed twice with DPBS and live-dead assays (Live/Dead mammalian cell viability/cytotoxicity kit L-3224, Life Technologies, Grand Island, NY) performed according to the manufacturer's protocol. This assay uses calcein AM (emission at 515 nm) for live cell stain and ethidium homodimer-1 (emission at 628 nm) as a dead cell stain. Following 30 min incubation with the fluorescent dyes, and rinsing with DPBS, the electrode slides were placed on the microscope stage for imaging. A Zeiss Axio Observer Z1 microscope, equipped with a CoolSNAP HQ2 CCD camera and Colibri 2 LED light source was used for image acquisition. A total of 32 areas (frames) were imaged along the full length of the electrode slide using a 5x lens. The average of three parallel rows was imaged for each electrode slide spaced 3 mm apart. Images were processed and analyzed with ZEN Pro2 (Zeiss) and Image J 1.48 software packages. All experiments were true replicates conducted in triplicate from separate cultures on the electrodes. Statistical analysis was performed using the SigmaPlot 12.5 software package (Systat Software, Inc.) Individual frame signals were spliced along the slide length resulting in a full slide live-dead cell image with subsequent averaging over 4 rows. The fraction of live cells was calculated by integrating the live (green calcein AM) fluorescence channel normalized by the total of live and dead (red ethidium homodimer) fluorescence channels.

### 2.3. Comet Assay

DNA strand breaks were measured by alkaline comet assay. Low melting point agarose (300 *μ*L, (LMPA), Trevigen, Inc., MD, USA Cat. No. 4250-050-02) was heated to 37°C and combined (ratio 1:10 volume fraction) with 30 *μ*L of a ≈2 × 10^5^ cells/mL suspension of thoroughly mixed cells collected as described above. Each well of a 20-well CometSlide (Trevigen, Inc., MD, USA Cat. No. 4252-200-01) was filled with 30 *μ*L of a thoroughly mixed cell/agarose suspension. The slides were placed in a 4°C refrigerator in the dark for 15 min to solidify. Slides were then immersed in 50 mL of pre-chilled lysis solution (3.2% w/w glycine, N,N′-1,2 ethanediylbis[N-(carboxymethyl)-, 1% w/w n-dodecylsarcosine, 1% poly(oxy-1,2 ethanediyl), α-[4-(1,1,3,3-tetramethylbutyl)phenyl]-ω-hydroxy-, Trevigen, Inc. Cat. No. 4250-010-01) and left at 4°C for 30 min to facilitate cell membrane and histone removal. After draining excess liquid, the slides were transferred to 50 mL of freshly prepared (same day) alkaline solution, (200 mmol/L NaOH, 1 mmol/L EDTA, pH > 13) and incubated at room temperature in the dark for 20 min to denature and unwind DNA. After the unwinding step, electrophoresis was performed at 4°C in the CometAssay ES tank filled with alkaline solution (Trevigen, Inc., MD, USA) at 21 V (1 V/cm) for 30 min. Slides were then rinsed with distilled water and fixed 5 min in 70% ethanol. Slides were dried and stained 5 min at 4°C with SYBR Green I (Trevigen, Inc., Cat. No. 4250-050-05) diluted 1 : 10 000 in 10 mmol/L Tris pH 7.5, 1 mmol/L EDTA, drained to remove excess staining solution and thoroughly dried at room temperature in the dark.

### 2.4. Microscopic Image Analysis

Slides were visualized by epifluorescence microscopy (Olympus System microscope, Model BH-2) equipped with the appropriate optical filter set for SYBR® Green I (excitation/emission wavelength, 460 nm and 560 nm respectively, Chroma, 49002 ET GFP) and a LUDL MAC 6000 automated stage and a Photometrics Snapcool HQ2 monochrome CCD camera using NIKON Elements software. Integrated intensities and Percent DNA in tail were determined using Image J (ver. 1.47v, NIH) and CometScore Pro (ver. 1.01.44, TriTek Corp., VA, USA) software utilizing the following equations:

(1) $$\begin{array}{c} \text{Total}\text{head}\text{intensity}I_{h}=\sum I_{h\left(x,y\right)}, \end{array}$$

(2) $$\begin{array}{c} \text{Total}\text{tail}\text{intensity}I_{t}=\sum I_{t\left(x,y\right)}, \end{array}$$

(3) $$\begin{array}{c} \%\text{DNA}\text{in}\text{tail}=\frac{100I_{t}}{I_{h}+I_{t}}, \end{array}$$

where *I*_*h*(*x*, *y*)_ and *I*_*t*(*x*, *y*)_ are the individual pixel intensities within the head and tail regions of the comet image. CometScore Pro is commercially available software which has been specifically developed to automate comet image analysis. We used the automated microscope system, controlled by NIKON elements software, in combination with the CometScore Pro software to quantify DNA damage as % DNA in tail in our cultured CHO cells after oxidative electrochemical treatment. All experiments were true replicates performed in triplicate originating from separate cultures on the electrodes.

## 3. Results

**Figure 1:**
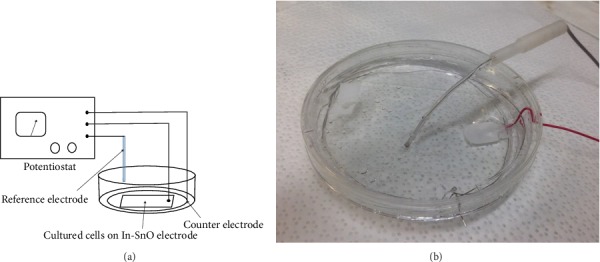
The electrochemical setup used to oxidize live mammalian cells (a) schematic diagram and (b) image of the electrochemical cell.

**Figure 2:**
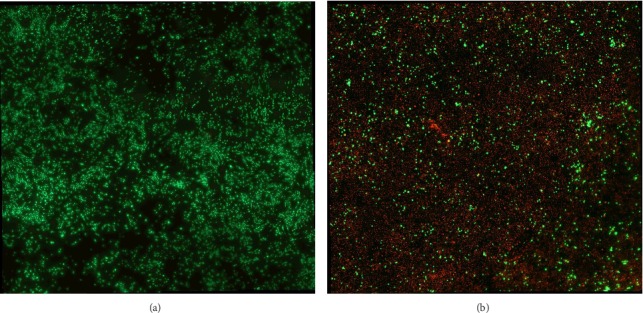
Fluorescent microscope images of the Live/Dead assay before (a) and after (b) the electrochemical treatment for 12 h at *E* = 1.0 V. The intracellular esterase activity by live cells is shown by the green fluorescent calcein dye. The loss of plasma membrane integrity of dead cells is shown by the red fluorescent ethidium homodimer. The calculated percentage of live cells before and after the electrochemical treatment is given in Table 1.

CHO cells were grown on a transparent InSnO_2_ electrode surface maintained for 12 h at a fixed potential. After treatment, the cells were analyzed while attached to the electrode by live/dead analysis or removed with trypsin from a separate electrode, treated in parallel, and analyzed by comet assay.  Figure 1(a) is a diagram of the electrode system with InSnO_2_ on glass serving as the working electrode.  Figure 1(b) is an image of the electrochemical cell. The cyclic voltammetry curve of the InSnO_2_ electrode, recorded in the growth medium, shows that the double layer charging region extends up to *E* = 1.5 V, thus avoiding gas evolution due to water electrolysis and cell detachment (Supplementary Figure S1),

Figure 2 shows typical fluorescent microscope images of the Live/Dead assay before (a) and after (b) electrochemical treatment after 12 h at *E* = 1.0 V. The live cells, in which the intracellular esterase activity is responsible for the green fluorescence of the calcein, are visible in the Figure. The loss of plasma membrane integrity in dead cells allows nuclear DNA staining by the red fluorescent ethidium homodimer. The fraction of live cells, calculated by integrating the calcein (green) fluorescence intensity normalized by the total of live and dead (ethidium bromide-red) fluorescence signal, before and after electrochemical treatment, is given in Table 1. Although the fraction of live cells in the untreated control is lower than expected for confluent cells grown in culture flasks, the gradual loss of cell viability is consistent with the electrode potential range that we observed previously with CHO cells grown in an electrochemical potential gradient [15].

**Figure 3:**
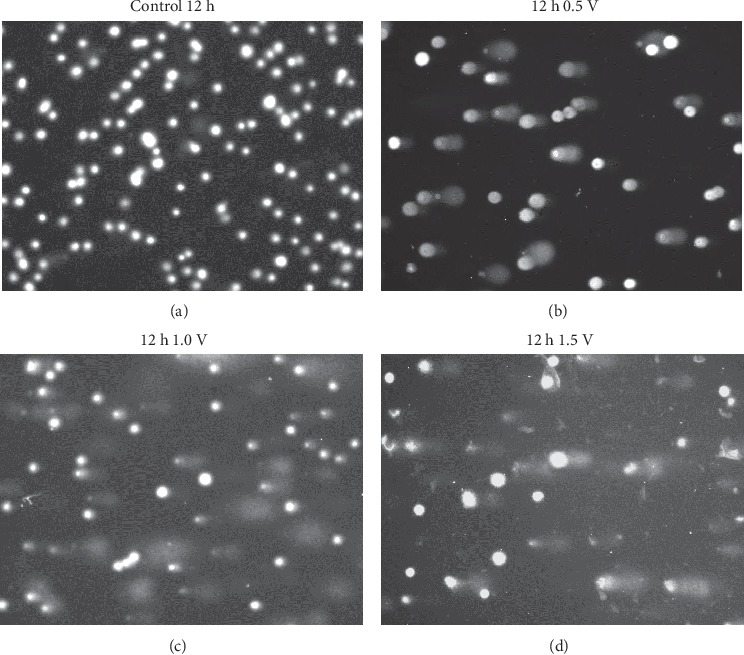
Fluorescent microscope images of representative comets (a) control, open circuit for 12 h (b) treated for 12 h at *E* = 0.5 V (c) treated for 12 h at *E* = 1.0 V (d) treated for 12 h at *E* = 1.5 V. The comet tails indicate the extent of DNA strand breaks.

Figure 3 shows representative fluorescent microscope images of cell comets before and after the electrochemical treatment at three electrode potential values for 12 h. The comet tail shape and size indicate the increase in DNA strand breaks with rising oxidation potential.

**Figure 4:**
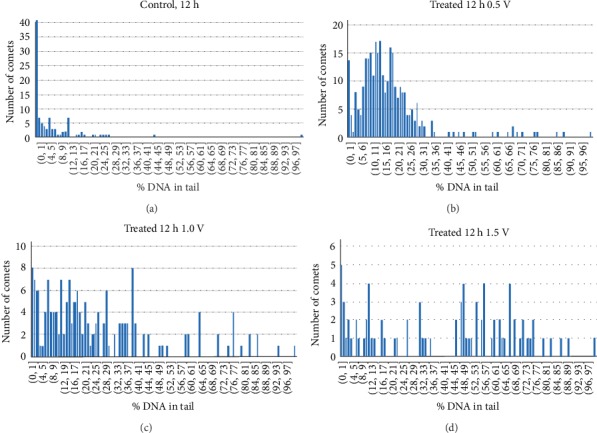
Representative histograms of the distribution of comets (a) control, open circuit for 12 h, *n* = 103 comets (b) treated for 12 h at *E* = 0.5 V, *n* = 302 comets (c) treated for 12 h at *E* = 1.0 V, *n* = 167 comets (d) treated for 12 h at *E* = 1.5 V, *n* = 82 comets. The number of comets within each bin is plotted as a function of % DNA in tail.

Representative histograms of the distribution of comets before and after treatment are shown in Figure 4. The histogram bin size was set equal to an estimated limit in resolution (1% error) in the measurement of % DNA in tail of individual comets, based on previous measurements of the average imaging reproducibility [10]. Histograms of all three replicate measurements (separate cultures on electrodes) are given in Supplementary Figure S2. We found that both the average level of the DNA damage (obtained by dividing the total sample % DNA in tail by the number of cells/comets) and the comet size distribution change with the treatment level. The *E* = 0.5 V treatment level yielded a relatively narrow distribution of comets with respect to % DNA that scales with the extent of DNA strand breaks. Essentially all of the comets were close to 30% DNA in tail. As expected, a higher oxidizing treatment level (*E* = 1.0 V) shifted comet size distribution towards a higher percentage of strand breaks (≈30% to 50% DNA in tail). At 1.5 V the distribution of comets became very diffuse with a majority of them having greater than 50% DNA in tail but almost half remaining less than 40% DNA in tail. This may be due to a population of cells that are able to maintain substantial DNA repair during this elevated level of treatment.

**Figure 5:**
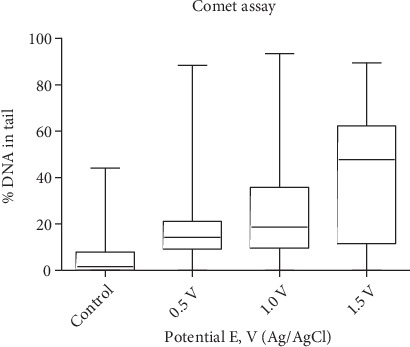
Box and whiskers plot of representative data shown in Figure 4. Boxes represent data within 25^th^ and 75^th^ percentiles. The horizontal line within each box represents the median value. Extended bars represent the max and minimum values.

**Figure 6:**
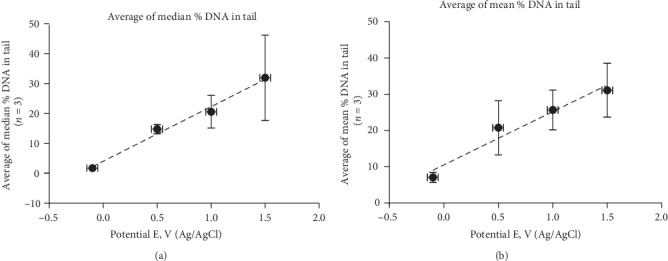
Comparison of comet analyses by median and means averages of % DNA in tail. (a) Plot of average of the comet assay medians as a function of increasing treatment level. Each data point at a given treatment level is the average of the medians of 3 independent comet distributions (histograms shown in Supplementary Figure S2). Error bars represent the standard deviation of the medians at each treatment level. (b) Plot of average of the comet assay means as a function of increasing treatment level. Each data point at a given treatment level is the average of the means of the same 3 independent comet distributions (histograms shown in Supplementary Figure S2). The vertical error bars represent the standard deviation of the medians or means at each treatment level. The horizontal error bars represent the small instrument uncertainty in the applied potential.

Figure 5 shows the box and whisker representation of the data shown in Figure 4. This type of plot displays both the median value and the heterogeneity in the population of cells after treatment. The plot confirms the increase in heterogeneity of the comets with increasing treatment levels, as indicated by the increasing vertical size of the boxes. The box and whisker plots of the replicate data is shown in Supplementary Figure S2.  Figure 6(a) is a plot of the average and standard deviation of the medians, as a function of increasing treatment potential of the replicate data. The increase in standard deviation, particularly at high electrode potential, is a reflection of experimental variation. The expression of the replicate data in terms of median values is important in that it is particularly sensitive to experimental variation in the distribution of the comets, particularly at high treatment levels.  Figure 6(b) is a plot of the average and standard deviation of the means of the replicate data. Although the median and the mean values are expected to be different with non-symmetrical distributions, both plots show essentially a linear increase in the percentage of damaged DNA with increasing oxidizing treatment level from *E* = 0.5 V to *E* = 1.5 V. A further oxidizing potential increase to *E* = 2 V for 12 h yielded extensive broken cells and debris that impeded comet analysis.

## 4. Discussion

The alkaline comet assay offers a sensitive detection of both single and double strand breaks. However, the inherent bio-variability of the cell's response to various steps of this procedure requires large numbers of cells to obtain a representative average. To obtain quality metrics, for most applications, about 100 cells are analyzed and this is practical only using an automated system for data collection and analysis [10, 17]. Another source of variability inherent in the comet assay is its multistep experimental procedure that contributes variation during lysis, electrophoresis, staining and imaging steps [9]. In addition, there is no consensus as to which single parameter is the best representative of the DNA damage extent (percent DNA in tail, tail length, tail moment, etc.) [12, 18]. We have chosen to express our data in terms of the percentage of DNA in tail (% DNA in tail), since this method yields the simplest direct estimate of the extent of DNA strand breaks, without distinguishing differences in the distribution of strand size, which can affect the shape of the tail (i.e., olive tail moment) [13, 18].

Various internal and external standards have been proposed to improve comet assay reproducibility [13, 14] and facilitate data comparability between laboratories. In the current study we have explored the electrochemical oxidation of surface attached CHO cells under potentiostatic conditions as a way to generate DNA damage reference materials for the comet assay. These measurements demonstrate that electrochemical oxidation of live cells, growing on an InSnO_2 _electrode surface, leads to reproducible DNA damage, as assessed by the comet assay, and could potentially be utilized for comet assay performance evaluation. In addition, the ROS generated by electrochemical oxidation may have unique properties at high oxidizing potential levels that could be relevant in the study of senescence and apoptosis.

A popular way to induce DNA damage *in-vitro* is to incubate the cells with chemical agents such as hydrogen peroxide. However, several factors inherent to chemical use are difficult to control and hamper the data comparability. The concentration of hydrogen peroxide is particularly difficult to quantify, primarily due to its instability in storage and in cellular media, which contains multiple reducing entities. Other widely used DNA damage inducing agents, such as etoposide, ethyl methane sulfonate or bleomycin also have the issues of accurate dosing due to difficulties with removal from cell preparations after treatment [19]. Alternatively, the exposure of cells to physical factors such as ionizing or UV radiation leads to a variety of DNA damage products which can be used as reference samples for the genotox assays. This requires specialized equipment and calibration of the radiation source. Radiation exposure of cells was reliably measured and dosing accurately controlled, by adjusting the exposure timing [14, 18]. Notably, there are no lingering DNA damage reactions following such treatments as opposed to residual chemical agents that diffuse into various cellular compartments [20]. In a similar fashion, the electrochemical treatment allows a well-controlled exposure of cells under a defined oxidative intensity level as prescribed by the electrode potential in a potentiostatic experiment.

During such exposure cells are oxidized directly and also react with electrochemically produced ROS resulting from water electrolysis. As in the case of ionizing radiation, electrode potential is easy to switch on and off, ensuring the accurate and reproducible dose control.

Previously, we showed that an electrochemical potential gradient can serve as a quantitative *in vitro* test platform for cellular oxidative stress in cultured mammalian cells [15]. In that study we used a live/dead assay to measure cell viability following their exposure to a range of the oxidizing potentials. We also have demonstrated that soluble genomic DNA is electro-oxidized on boron doped diamond electrodes under potentiostatic conditions [16]. Our GC/MS/MS measurements of purified calf thymus DNA showed that base lesions (8-hydroxyguanine, 8-hydroxyadenine and 5-hydroxy-5-methylhydantoin) were produced during electrochemical treatment at *E* = 2.0 V for 1 h [16]. Also, in an earlier study, using capillary electrophoresis, we found extensive strand breakage in calf thymus DNA when exposed for 1 h at *E* = 3.0 V and in Poly A and Poly G nucleotides exposed 1 h at *E* = 1.0 V [21]. These studies show that the production of significant DNA damage under physiological conditions in this electrode potential range is consistent with our current studies of mammalian cells.

In the current investigation, we used the comet assay to examine the extent of DNA damage produced in live cells at increasing levels of oxidative stress exerted by the working electrode potential. Our use of histograms to evaluate the effect of increasing levels of electrochemical treatment reveals a population of cells that apparently are able to maintain DNA repair at high treatment levels. This type of plot, using a bin size at the measurement resolution of imaging % DNA in tail for individual comets, yields a complete picture of the heterogeneity in the distribution of comet size. As shown in Figure 4, at treatment levels of *E* = 1.0 V and *E* = 1.5 V, a substantial percentage of the cells are able to maintain DNA damage levels approximating that observed at *E* = 0.5 V. This explanation seems likely given that about 30% of the cells remain viable at *E* = 1.0 V by the Live/Dead assay (Table 1). Since the asynchronous culture of cells was treated over a period of 12 h, they would be equally affected by treatment during their replication cycles. However, some cells in the culture may be replicating faster than others and those cells may be more sensitive to damage. If instead the heterogeneity was due to the cells exposed to a non-homogeneous environment (i.e., non-uniform potential on the electrode surface) a wider distribution of comets would also be expected in the histograms at the lower *E* = 0.5 V treatment level. Instead the distribution of comets is more homogeneous at 0.5 V compared to the higher levels of treatment (Figure 4). In this regard, *E* = 0.5 V may be an optimal range for use to produce a reference material. The box and whiskers plot is particularly helpful to compare the extent of heterogeneity in the population of cells after treatment (Figure 5). However, a more in-depth analysis of the cell populations with increasing levels of treatment, using assays for apoptosis and senescence, may be required to elucidate the biological reasons for this heterogeneity.

To assess the repeatability of the electrochemical oxidation, including any subsequent variations in the comet assay, all three independent sets of treated and analyzed cells were compared (Supplementary Figure S2). Despite the observed heterogeneity and experimental variation, particularly at the higher treatment levels, when the three sets of medians and the means of the individual histograms were averaged, both the plot of the average median % DNA in tail (Figure 6(a)) and the plot of the average mean % DNA in tail (Figure 6(b)) showed a linear dependence within the range of 0 V to 1.5 V. The standard deviations of the individual medians indicated a continuous increase in experimental variation with treatment level. At *E* = 0.5 V, the variation of the means is much greater than that of the medians, which demonstrates the high sensitivity of the mean in detecting the high % DNA in tail outliers that can appear at this treatment level. In addition, at *E* = 0 V, the 5% higher value of the average of mean % DNA in tail, plotted in Figure 6(b), indicates that the mean is more sensitive to data asymmetry. This observation supports the concept that a low level of DNA damaging events, measured here in strand breaks, occurs as background in the absence of applied genotoxins (no threshold). This is shown in the histogram in Figure 4(a) where a substantial number of cells containing DNA strand breaks at zero oxidative bias is evident and in the average of mean plot vs applied potential, where the asymmetry in the distribution is emphasized (Figure 6(b)).

Although purified DNA containing various levels of damage can be stored for extended periods, the stability of the electrochemically treated mammalian cells during cold storage was not determined here. As with other DNA damaging methods, the effect of active repair enzymes in electrochemically treated cells under various storage conditions will need to be examined. In addition to stability during storage, a reference material would require identical aliquots from a single batch of treated cells. The electrochemical method could be practical to produce reference materials, particularly at the lower treatment level (*E* = 0.5 V), where homogeneity and repeatability can be achieved. Multiple preparations of about a million cells each, at this treatment level, could easily be combined into a single large batch of several million cells for storage and shipment of identical samples to different research groups for comparison between labs. The box and whiskers and mean analysis can be used during this process for quality control and to eliminate any aberrant preparations from being included in the batch. The resulting reference material aliquots could then be comet assayed in parallel with test materials, as a quality control of the procedure. However, for use of the electrochemical system to generate custom samples on site as needed for a reference would require low treatment levels (i.e., *E* = 0.5 V) where repeatability is optimal. For results to be comparable between different laboratories, the electrochemical system, cell type and treatment conditions would need to be completely specified. Furthermore, measurements by alternative assay methods may be needed to verify the actual mass percentage of damaged DNA.

## 5. Conclusions

Treating CHO cells grown on an indium tin oxide electrode by electrochemical oxidation is an efficient method to produce DNA damaged cells under well-controlled conditions. This approach has potential use in preparing cellular reference materials for the comet assay as well as other bio-analytical applications. The appeal of this cellular treatment method is that it does not require complicated or hazardous equipment and samples for DNA damage assay calibration can conveniently be prepared. However, for these products to be used as reference materials their complete characterization and analysis of stability will be required.

## Figures and Tables

**Table 1:** Live/Dead analysis of cells before and after treatment.

Treatment	Fraction live
Control, 12 h	0.64 ± 0.29
12 h *E* = 0.5 V	0.53 ± 0.12
12 h *E* = 1.0 V	0.30 ± 0.15

Errors are standard deviations of three independent measurements (*N* = 3).

## Data Availability

The supporting data is available in the submitted manuscript and the supplementary materials files.

## Supplementary Materials

Supplementary Materials Figure S1: cyclic voltammetry of the indium tin oxide electrode, recorded in complete cell growth medium. Scan rate is 10 mV/s. Figure S2: reproducibility of comet assay. Histograms of comet data resulting from separate cultures and electrochemical treatments. (a) Control, open circuit for 12 h. (b) 12 h at *E* = 0.5 V. (c) 12 h at *E* = 1.0 V. (d) 12 h at *E* = 1.5 V. Treatment time and potential and number of comets counted are given above each histogram. Box and Whisker Plots of the individual data sets are shown for each treatment level, which illustrates the median, the 25 and 75 percentiles and the outliers for each electrochemical treatment. *N* = 3 independent experiments are shown for each treatment level.
