# Supplementary material for: Cellular Reference Materials for DNA Damage Using Electrochemical Oxidation
Source: J Nucleic Acids. 2020 Jan 30;2020:2928104. doi: 10.1155/2020/2928104 (PMC7212329; doi:10.1155/2020/2928104)
Supplement: Supplementary Materials — Figure S1: cyclic voltammetry of the indium tin oxide electrode, recorded in complete cell growth medium. Scan rate is 10 mV/s. Figure S2: reproducibility of comet assay. Histograms of comet data resulting from separate cultures and electrochemical treatments. (a) Control, open circuit for 12 h. (b) 12 h at E = 0.5 V. (c) 12 h at E = 1.0 V. (d) 12 h at E = 1.5 V. Treatment time and potential and number of comets counted are given above each histogram. Box and Whisker Plots of the individual data sets are shown for each treatment level, which illustrates the median, the 25 and 75 percentiles and the outliers for each electrochemical treatment. N = 3 independent experiments are shown for each treatment level. [file 2928104.f1.pdf]

Supplementary Figure S1

Cyclic Voltammetry of the Indium Tin Oxide Electrode

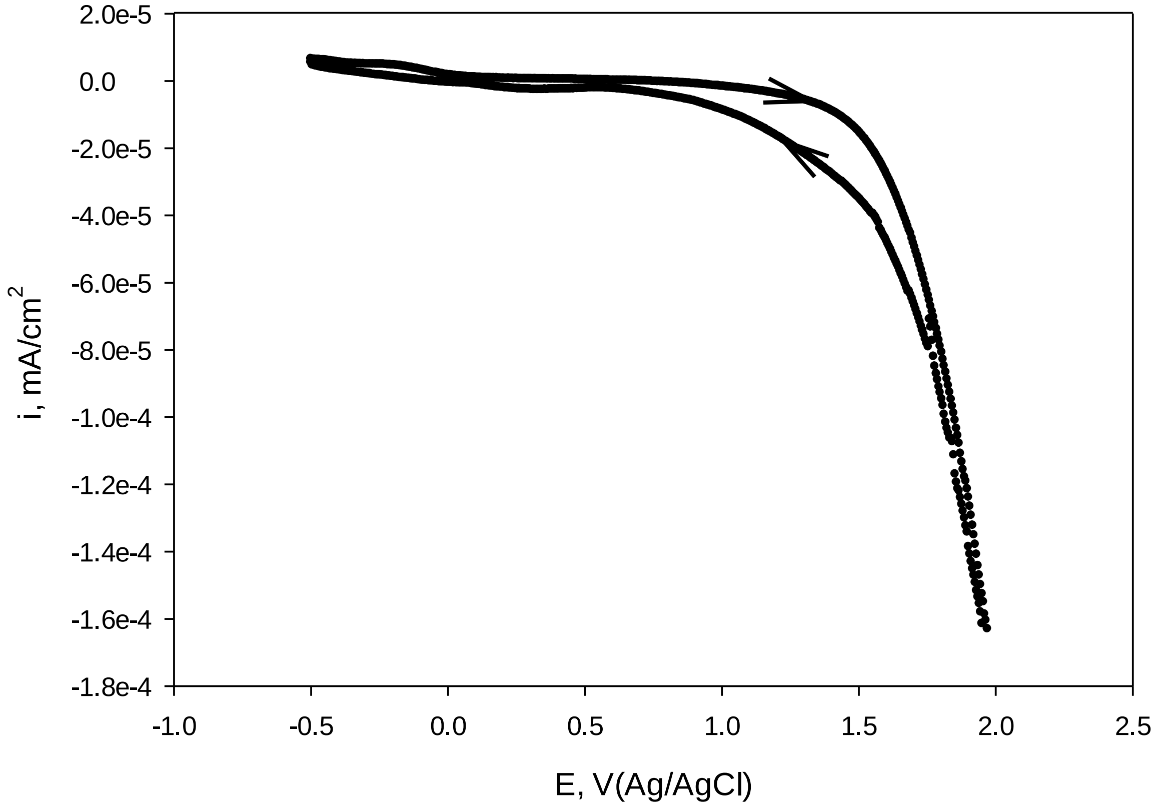

Supplementary Figure S2

Repeatability of Electrochemical treatment and Comet Assay

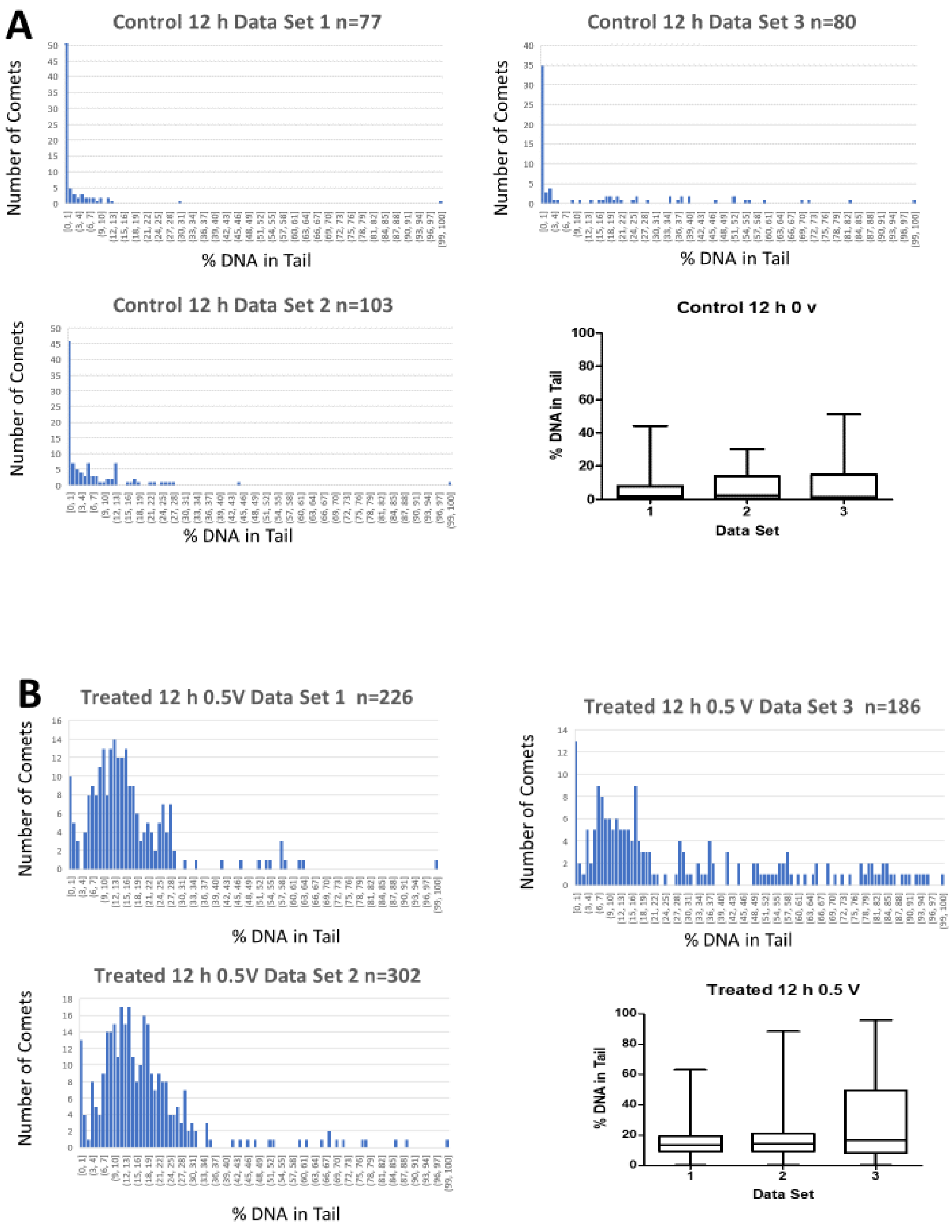

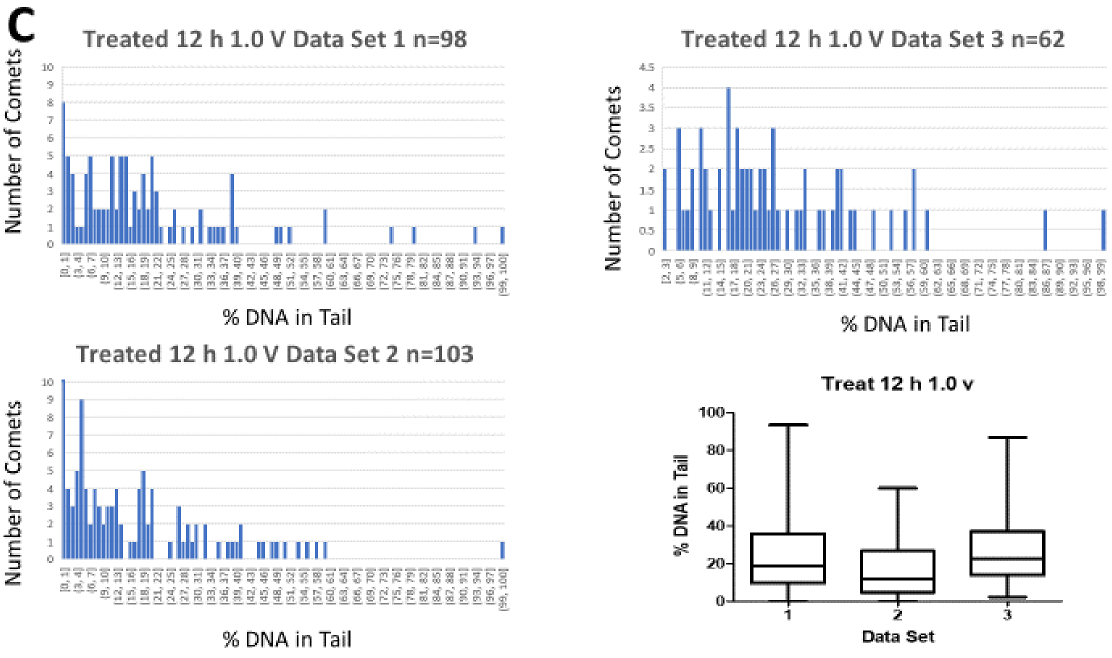

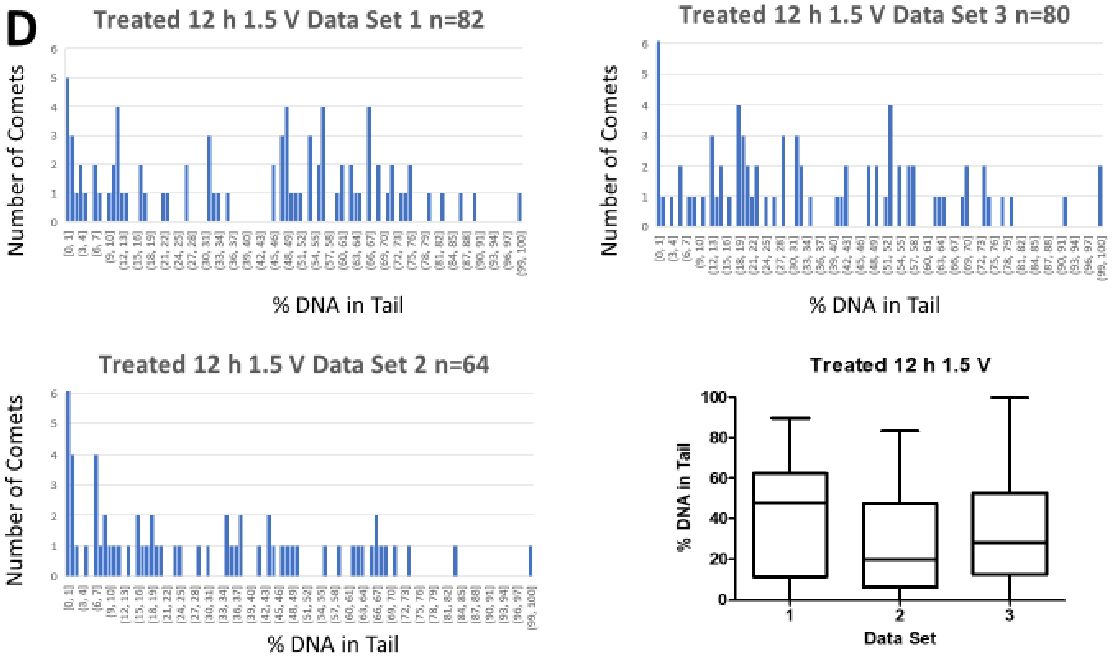

Three Independent measurements at each treatment level (N=3)
